# Discovery of Traditional Chinese Medicine Prescription Patterns Containing Herbal Dosage Based on Multilevel Top-K Weighted Association Rules

**DOI:** 10.1155/2022/5466011

**Published:** 2022-05-25

**Authors:** Xiaolin Zhu

**Affiliations:** ^1^School of Computer Science, China West Normal University, Nanchong 637002, China; ^2^Internet of Things Perception and Big Data Analysis Key Laboratory of Nanchong, Nanchong 637002, China

## Abstract

In traditional Chinese medicine (TCM), drug dosage is an important part of the prescription. Different doses of the same drug can have varying curative effects. Doctors must determine the drug combination and dosage in clinical practice based on the patient's symptoms and treatment efficacy. Existing studies on the prescription pattern of TCM on the treatment of osteoporosis only analyze the frequency that a certain drug combination is used, without considering the treatment efficacy or drug dosage. As a result, we searched for and recorded existing literature on randomized controlled trials of TCM treatment of osteoporosis, calculated weights based on the treatment efficacy of the prescriptions used in the randomized controlled trials, and created the TCM weighted transaction database. Then, a new multilevel Top-*K* weighted association rule algorithm is proposed to obtain effective prescription patterns that include drug dosages, which can assist doctors in clinical practice in choosing a combination of drugs to form a prescription with good curative effects.

## 1. Introduction

The association rule algorithm is used to mine drug usage rules from a large number of traditional Chinese medicine (TCM) prescriptions, thereby providing doctors with useful information about core drugs and prescriptions [1]. This assists doctors not only in formulating diagnoses and treatment strategies but also in choosing appropriate clinical treatments [2]. Yang et al. [3] found that Dipsaci Radix is the most commonly used drug from the prescriptions of TCM treatment of threatened abortion recorded in the literature. The most common two-drug combination is Cuscutae Semen and Dipsaci Radix, and the most common three-drug combination is Cuscutae Semen, Herba, and Dipsaci Radix. These rules aid in comprehending the theory of threatened abortion in TCM treatment. Chen et al. [4] analyzed the prescription of TCM treatment of acne and found that Qing-Shang-Fang-Feng-Tang has a support of 31.2%, which is the most commonly used compound medicine, while Zhen-Ren-Huo-Ming-Yin is often used with Forsythia suspensa to form the most commonly used drug combination. Peptic ulcer disease is a common disease. Huang et al. [5] discovered that in TCM, the most commonly used compound medicines and single drugs to treat the disease are Ban-Xia-Xie-Xin-Tang and Os Sepiae. The most commonly used 5-drug combinations are Ban-Xia-Xie-Xin-Tang, Os Sepiae, Rhizoma Corydalis, Bulbus Fritillariae Thunbergii, and Fructus Toosendan.

Osteoporosis is a group of bone diseases caused by various reasons. It is distinguished by a reduction in the amount of bone tissue per unit volume and thinning of the bone cortex, which results in limb pain, spinal deformity, and even fractures. Similar disease names in Chinese medicine include osteopenia and bone atrophy. Chinese medicine has long used syndrome differentiation to diagnose and treat osteoporosis, with good results and a large number of prescriptions. Numerous existing studies shed light on prescriptions and medication rules derived from these prescriptions. Shih et al. [6] used the association rule algorithm to understand the prescriptions for the treatment of osteoporosis in the National Health Insurance Research Database and found the hidden prescription patterns: Du-Huo-Ji-Sheng-Tang and Cortex Eucommiae are the most commonly used compound medicine and single drug. Radix Dipsaci and Cortex Eucommiae are the most commonly used 2-drug combinations, followed by Cortex Eucommiae and Radix Achyranthis. Zhang et al. [7] searched the PubMed and Chinese National Knowledge Infrastructure (CNKI) databases for research on TCM treatment of osteoporosis and then analyzed 33 TCM prescriptions. They found that the most commonly used drug for TCM treatment of osteoporosis is Rehmannia glutinosa Libosch, which occurred 14 times in the literature, followed by Epimedium brevicornu Maxim, which was used 11 times.

However, these studies use traditional association rule algorithms, such as Apriori [8], to mine the most commonly used drug combinations while only considering the frequency of drugs in the prescription, and neglect the treatment efficacy and drug dosage. When the dosage of drugs is different, they tend to produce different curative effects [9]. Therefore, these mined prescription patterns cannot provide effective assistance to the doctors in clinic. To solve these problems, we first searched and recorded the research literature related to randomized controlled trials of TCM treatment of osteoporosis. These types of literature have recorded the drugs, and their doses provided in prescriptions that were used in the randomized controlled trials. The total number of samples in the treatment group and the control group, and the number of samples in which the treatment is effective have also been recorded. The prescription weight was determined according to the number of effective samples in the treatment and the control group, and the TCM weighted transaction database (TCMWTD) was generated. Then, a new multilevel Top-K weighted association rule algorithm (MTWSR) is proposed to recommend prescriptions based on curative effects and obtain effective prescription patterns that include drug dosages, which can help doctors choose a combination of drugs to form a prescription with good curative effects in clinical practice.

## 2. Preliminaries

### 2.1. Weighted Association Rules

Let *P* is composed of *k* items, and then, *P* is a pattern or itemset of length *k*, that is, *k*-patterns or *k*-itemsets. Support(*P*) is the number of transactions that include *P* in the transaction database divided by the number of all transactions in the transaction database [8]. If Support(*P*) is larger than the minimum support threshold *ξ*, then *P* is defined to be frequent itemset. The traditional association rule algorithm mines frequent itemsets.

In practical applications, the importance of a certain factor is determined by the very scenario to which the algorithm is applied. For example, because different prescriptions and drugs have different curative effects, doctors prefer to refer to drug combination patterns derived from prescriptions that have good curative effects. As a result, Ramkumar et al. [10] proposed the concept of weighted association rules, and the weighted transaction database (WTD) is constructed after assigning corresponding weights based on their importance. The term “weighted support” is defined by Tao et al. [11]. The weighted support of itemset *P* is recorded as WS(*P*) and is equal to the sum of the weights of all transactions containing *P* in the WTD divided by the sum of the weights of all transactions in the WTD. If WS(*P*) is greater than the minimum weighted support threshold, then *P* is a frequent weighted itemset or pattern (FWI). The FWI of length *k* is denoted as k-FWI, and the frequent weighted pattern set is denoted as FWIs. Mining FWIs is the main task of the weighted association rule algorithm.

### 2.2. Meta-Analysis and Its Application in Traditional Chinese Medicine

Meta-analysis is a systematic analysis and comprehensive evaluation of multiple independent research results for the same clinical problem to produce scientific analysis results. There have been numerous meta-analyses of randomized controlled trials of TCM. Pang et al. [12], for example, conducted a meta-analysis of the retrieved randomized controlled trials on the use of TCM. In the treatment of diabetic peripheral neuropathy, Huangqi Guizhi Wuwu Decoction (HGWWD) is used. They concluded that the usage of HGWWD has a significant effect on improving diabetic neurological symptoms and nerve conduction velocity. In the process of meta-analysis, weights are assigned to the prescriptions according to the curative effect of the prescriptions used in each randomized controlled trial [13]. The corresponding sample sizes of the treatment and control groups in the randomized controlled trial are set to *N*_1_ and *N*_2_, respectively. The number of samples with effective treatment is set to *E*_1_ and *E*_2_, respectively. Then, the formula for calculating the prescription weight *W* used in this experiment is defined as follows:

(1) $$\begin{array}{c} W=\frac{1}{\left(1/E_{1}\right)-\left(1/N_{1}\right)+\left(1/E_{2}\right)-\left(1/N_{2}\right)}. \end{array}$$

### 2.3. Multilevel Association Rules

Sometimes there is a hierarchical relationship between the items of the transaction. When this happens, the support of the level-1 item is equal to the sum of the support of its corresponding level-2 items. In supermarket sales records, for example, bread is divided into wheat and white, with a hierarchical relationship between them. Bread support is equal to the sum of wheat bread and white bread support. To mine different levels of association rules, Han and Fu [14] proposed the multilevel association rule algorithm ML_T2L1, which sets a different minimum support threshold for each level of association rules, and adopts a strategy similar to the Apriori algorithm by first repeatedly scanning the transaction database to mine the most level-1 association rules, filtering the transaction database according to the most level-1 association rules, and then mining the level-2 association rules. However, the algorithm is inefficient, and determining an appropriate minimum support threshold for each level of association rules is extremely difficult. It is necessary to improve the algorithm.

## 3. The Proposed Algorithm

In the process of mining prescription patterns with good curative effects, it is difficult to accurately estimate the minimum support threshold *ξ*. If *ξ* is set too small, a large number of prescription patterns will be mined, and if it is set too large, useful prescription patterns will be lost. Based on the *N*-most interesting itemsets [15], we change *ξ* to easily set parameters *K*, *N*, and *n*. Among them, *K* is the length of the longest level-1 FWIs mined, *N* is the number of level-1 FWIs of each length, and *n* is the number of level-2 FWIs corresponding to each level-1 FWI.

**Definition 1 .**

(the N-most k-FWI). When all k-FWIs are arranged in descending order of WS, if the weighted support of the *N*th k-FWI is WS_*N*_, then all k-FWIs with WS greater than WS_*N*_ form the N-most k-FWI.

**Definition 2 .**

(the Top-K FWI). When 1 ≤ *k* ≤ *K*, all N-most k-FWIs form Top-K FWI.

**Definition 3 .**

(the multilevel Top-K FWI). Composed of level-1 Top-K FWI and its corresponding level-2 Top-K FWI.

The multilevel Top-K weighted association rules mining algorithm (MTWSR) proposed in this study first assigns corresponding weights to the prescriptions based on the treatment efficacy, then constructs the Multilevel Weighted Prefix Tree (MWP-tree), and mines the level-1 Top-K FWI to get the best combination of drugs. Finally, it mines level-2 Top-K FWI to get the optimal dosage of drug combinations.

### 3.1. Determination of Prescription Weights

First, the absolute weight of each prescription is calculated according to formula (1), and then, the absolute weight of each prescription is divided by the sum of the absolute weights of all prescriptions to obtain its weight. All TCM prescriptions and their weights form the TCM weighted transaction database (TCMWTD).

**Example 1 .**

The prescriptions used in the five randomized controlled trials of TCM treatment of osteoporosis are shown in Table 1. The drug is recorded in front of the *∗* symbol, and the dosage of the drug follows the *∗* symbol. The efficacy of each prescription is shown in Table 2. In the randomized controlled trial using the first prescription, among the 100 patients in the treatment group, 91 were effectively treated; while in the control group, 82 patients were effectively treated. Therefore, according to formula (1), the absolute weight of the prescription is as follows:

(2) $$\begin{array}{c} W=\frac{1}{\left(1/91\right)-\left(1/100\right)+\left(1/82\right)-\left(1/100\right)}=314.1. \end{array}$$

With the above, the sum of the absolute weights of all prescriptions is calculated to be 1010.74, and the weight of the first prescription is 314.1/1010.74 = 0.31.

This study focuses on the prescription of TCM, and the drugs and dosage contained in the prescription are the important items. The drug and the dosage have a hierarchical relationship between them, and the weighted support of the drug is equal to the sum of the weighted support of the drug dosage.

### 3.2. Constructing the MWP-Tree

**Definition 4 .**

(Item-Index). Arrange all drugs in descending order of their weighted support, and the sequence number obtained is the item-index of the drug. The item-index of the drug with the highest weighted support is 1.

**Definition 5 .**

MWP-tree consists of a HeaderTable and the multilevel prefix tree. The HeaderTable stores the item-index of all drugs, and each unit consists of 5 domains: item-names, item-index, dosage, weighted support, and node-links. Item-names stores the drug's name, Item-index stores the drug's item-index; dosage stores the drug dosage in grams; and weighted support stores the drug dosage's weighted support. Node-links store nodes in the prefix tree that have the same item-index. The root node of the multilevel prefix tree is Root, and the prefix tree node consists of 6 fields: item-index, dosage, weighted support, parent-pointer, child-pointer, and node-links. The parent-pointer and child-pointer fields point to the parent node and child node, respectively.

First, the MTWSR algorithm scans TCMWTD, calculates the weighted support of all drugs, sorts them according to the weighted support, assigns an item-index to each drug, and generates a *HeaderTable*. Next, the algorithm scans the TCMWTD again, maps the drugs in each transaction to item-index, sorts them in descending order of weighted support, and then inserts the transactions into the MWP-tree in turn. The process is as follows:

When inserting transaction *T*_1_, insert the item-index and the dosage of the first drug of *T*_1_ with *Root* as the current node, which is labeled as *II*_1_ and *DS*_1_, respectively. Check if *Root* has child nodes whose item-index and dosage domain values are equal to *II*_1_ and *DS*_1_. If this is the case, the child node becomes the current node, and its *weighted support* domain value is added to *T*_1_'s weight. Otherwise, create a new child node, set its *item-index* and *dosage* domain values to *II*_1_ and *DS*_1_, respectively, and set its *weighted support* domain to *T*_1_'s weight. Use this new child node as the current node. At the same time, connect the *node-links* of the current node to other nodes that record *II*_1_ and *DS*_1_ in the MWP-tree, and the *node-links* field of the unit that records *II*_1_ and *DS*_1_ in the *HeaderTable* points to the current node. Then, from the current node, insert the item-index and the dosage of *T*_1_'s second drug, which are labeled *II*_2_ and *DS*_2_, respectively, and see if the current node has child nodes with *item-index* and *dosage* domain values equal to *II*_2_ and *DS*_2_. If so, the child node becomes the current node and its *weighted support* domain value is added to the weight of *T*_1_. Otherwise, create a new child node, set its *item-index* and *dosage* domain values to *II*_2_ and *DS*_2_, respectively, and its *weighted support* domain to the weight of *T*_1_, and make this new child node the current node. Then, in *T*_1_, insert the item-index and the dosage of the remaining drugs using the same method. With this procedure, insert all transactions in TCMWTD.

**Figure 1:**
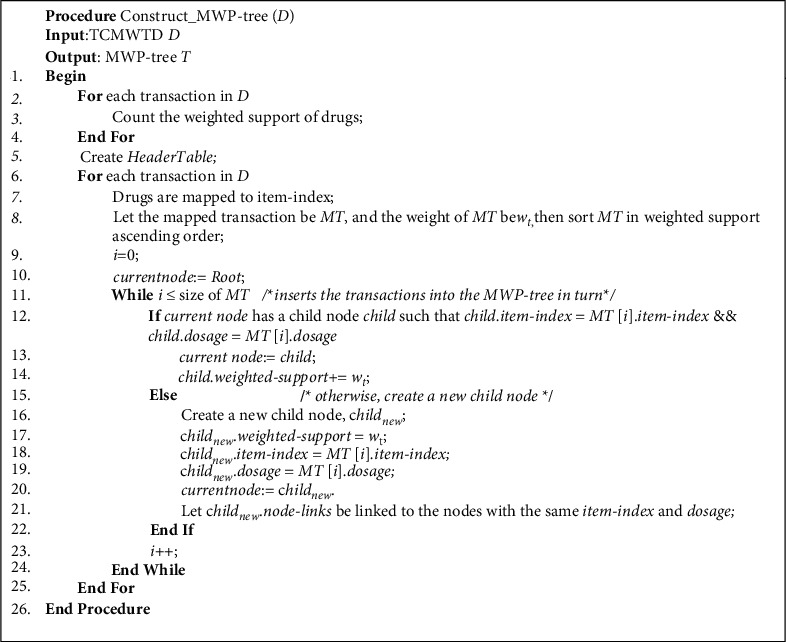
MWP-tree construction algorithm.

The pseudocode of the algorithm for constructing the MWP-tree is shown in Figure 1.

The following example details how to construct MWP-tree:

**Example 2 .**

**Figure 2:**
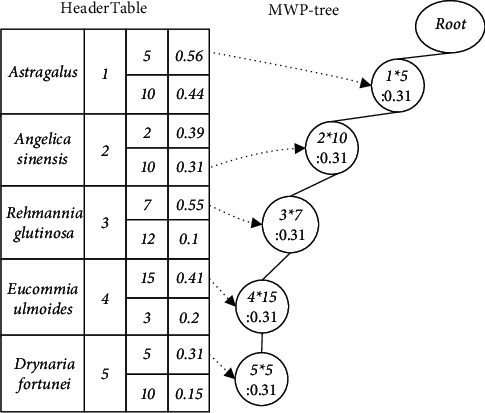
An example of an MWP-tree state: inserting the first transaction.

**Figure 3:**
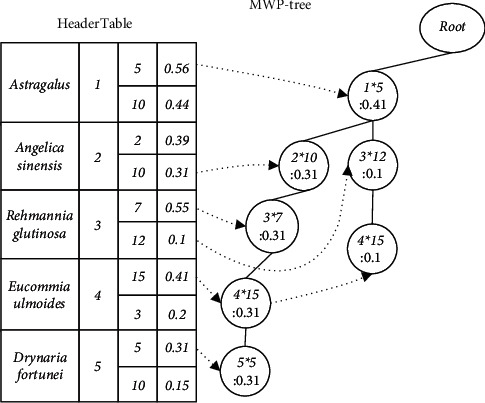
An example of an MWP-tree state: inserting the second transaction.

**Figure 4:**
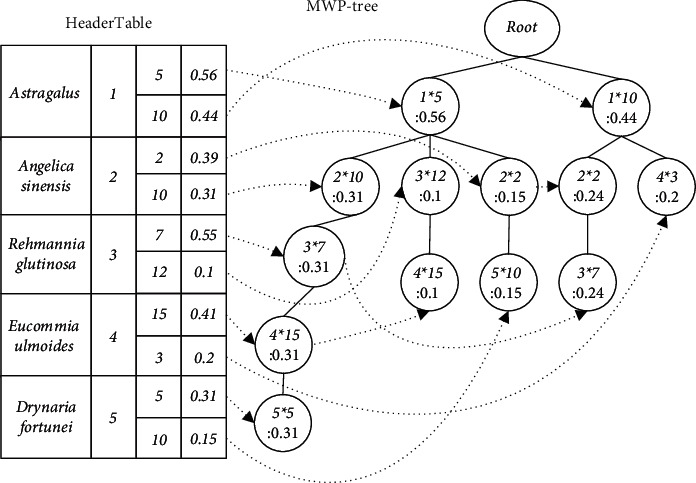
An example of an MWP-tree state: inserting all transactions.

The MWP-tree is constructed from the TCMWTD shown in Table 1. First scan the TCMWTD, calculate the weighted support of each item (drug), and arrange them in descending order: Astragalus: 1.0, Angelica sinensis: 0.7, Rehmannia glutinosa: 0.65, Eucommia ulmoides: 0.61, and Drynaria fortunei: 0.46. Their item-indexes are as follows: 1, 2, 3, 4, and 5, respectively. Thereafter, in descending order of weighted support, initialize the MWP-tree, generate the HeaderTable, scan the TCMWTD again, map the items in each transaction to the corresponding item-index, and insert the MWP-tree. The first transaction after mapping and sorting is {1 ∗ 5 g, 2 ∗ 10 g, 3 ∗ 7 g, 4 ∗ 15 g, 5 ∗ 5 g}. Insert the MWP-tree from 1 ∗ 5 g. The current node Root has no child nodes, so a new child node (1 ∗ 5: 0.31) is generated and used as the current node with the item-index domain value set to 1 and the dosage domain value set to 5. The weighted support domain value is equal to the weight of the first transaction: 0.31. Next, insert 2 ∗ 10 g. As the current node does not have any child nodes either, a new child node (2 ∗ 10: 0.31) is generated and used as the current node. Using this method, one can insert all the item-indexes and their dosage of the first transaction, as shown in Figure 2, where “:” is followed by WS. The second transaction after mapping and sorting is {1 ∗ 5 g, 3 ∗ 12 g, 5 ∗ 15 g}. Insert the MWP-tree from 1 ∗ 5 g as shown in Figure 3. The MWP-tree after inserting all transactions is shown in Figure 4.

After the construction of the MWP-tree is completed, the level-1 Top-K FWI is mined without considering the dosage.

### 3.3. Mining Level-1 Top-K FWI

**Definition 6 .**

The mining result list (MRL) is used to save the multilevel Top-K FWI. There are *N* weighted frequent itemsets with length *k* (1 ≤ *k* ≤ *K*), so MRL has *K*×*N* units in total. Each unit has 1 field to store level-1 FWI, and *n* fields to store level-2 FWI. In MRL, the minimum weighted support of all level-1 FWIs is recorded as *δ*^1^, and the minimum weighted support of all level-1 k-FWIs is recorded as *δ*_*k*_^1^. The minimum weighted support of all level-2 k-FWI corresponds to level-1 k-FWI *β*, which is recorded as *δ*_*k*_^2^(*β*). Their initial values are all 0.

**Definition 7 .**

(Level-1 MWP-tree). An MWP-tree where the dosage field value of all tree nodes is empty.

Suppose the process of mining level-1 Top-K FWI is MWP-growth-1(), which has two input parameters: MWP-tree, MT; a prefix, pf, and its initial value is null. The execution process is as follows:

Starting from the unit with the highest WS in the HeaderTable of MT, visit each unit in turn until the WS of the unit is smaller than *δ*^1^. Using the item-index *a*_*i*_ saved in each unit, access each tree node in the MT that saves *a*_*i*_ through node-links, and then traverse the path from these nodes to the Root. Assuming that the unit storing *a*_*i*_ is connected to the tree node *L* through node-links, then pf and *a*_*i*_ are combined to form a new prefix pf′ and stored in MRL. Check if the node-links of *L* are empty:

- If it is empty, there is only one path from *L* to Root. Access all nodes on the path through the parent-pointer, combine pf′ and item-index in these nodes to form level-1 k-FWIs (1 ≤ *k* ≤ *K*), and let the WS of these new patterns be equal to WS of *L*. Use the procedure Insert-MRL() to store these new patterns in the MRL.
- If it is not empty, there are multiple paths from *L* to Root. Combine the item-index on each path into a level-1 conditional pattern, use the procedure Construct_MWP-tree(), and construct the level-1 conditional MWP-tree CMT from these conditional patterns. Then, iteratively call the procedure MWP-growth-1() with CMT and pf′ as input parameters.

**Figure 5:**
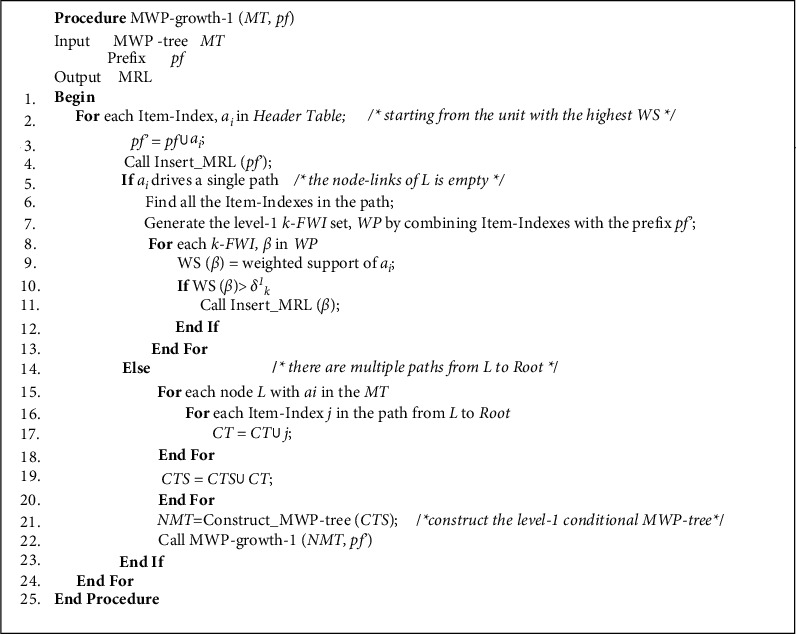
Algorithm for MWP-growth-1.

**Figure 6:**
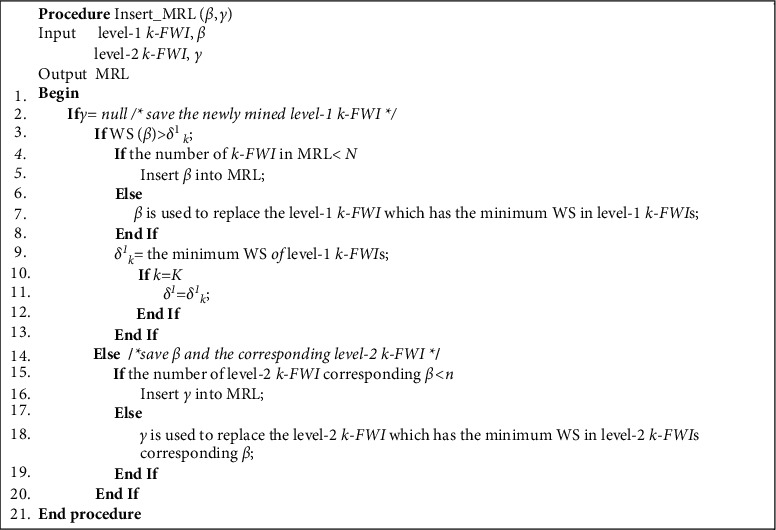
Algorithm for Insert_MRL.

Insert_MRL() saves the newly mined level-1 k-FWI, *β*, and its execution process is as follows:

If WS(*β*) > *δ*_*k*_^1^, first determine whether the number of k-FWI saved in MRL is smaller than *N*. If it is, insert *β* directly into MRL; otherwise, replace the k-FWI with the smallest WS in MRL with *β*, let *δ*_*k*_^1^ = the smallest WS that appears in level-1 k-FWI. At the same time, if *k* = *K*, then *δ*^1^ = *δ*_*k*_^1^. The pseudocodes of the process MWP-growth-1() and Insert_MRL() are shown in Figures 5 and 6.

**Example 3 .**

Set *K* = 3, *N* = 3, and mine the level-1 Top-K FWI from the MWP-tree shown in Figure 4. There are two paths from the first unit of the HeaderTable to Root: {1 ∗ 5 : 0.56}, {1 ∗ 10 : 0.44}, so FWI: {1 : 1} is inserted into MRL. Then, since there are 3 paths to Root from the unit where item-index “2” is located, 3 level-1 conditional patterns are generated: <1 : 0.31>, <1 : 0.15>, and <1 : 0.24>. The resulting level-1 condition MWP-tree has only one node that saves “1,” and FWIs can be obtained as follows: {2 : 0.7} and {2, 1 : 0.7} are inserted into the MRL. There are 3 paths from the unit where item-index “3” is located to Root, and the condition patterns of prefix “3” can be obtained: <2, 1 : 0.31>, <1 : 0.1>, and <2, 1 : 0.24>. The generated level-1 condition MWP-tree has only 1 path, and FWIs can be obtained as follows: {3 : 0.65}, {3, 1 : 0.65}, and {3, 2, 1 : 0.55}. The MRL after level-1 Top-K FWI mining is shown in Table 3.

After completing the level-1 Top-K FWI mining, start mining the level-2 Top-K FWI.

### 3.4. Mining Level-2 Top-K FWI

Suppose the mining level-2 Top-K FWI process is MWP-growth-2(), which has 3 input parameters: MWP-tree labeled MT; a level-1 prefix, labeled pf_1_; and a level-2 prefix, labeled pf_2_. The execution process is as follows:

Starting from the highest unit of WS in the HeaderTable of MT, visit each unit in turn downward. Assuming that the item-index field value saved by the unit is *a*_*i*_ and the dosage field value is *d*_*i*_, then *a*_*i*_ and pf_1_ are combined to form a new level-1 prefix pf_1_′, and *d*_*i*_ and pf_2_ are combined to form a new level-2 prefix pf_2_′. First, determine whether pf_1_′ is the level-1 k-FWI in the MRL, and if so, insert pf_2_′ as the level-2 k-FWI corresponding to pf_1_′ into the MRL. Access each tree nodes saved *a*_*i*_ and *d*_*i*_ via node-links and follow the path from these nodes to Root. Let *L* be the unit storing *a*_*i*_ and then determine whether *L*'s node-links are empty:

- If it is empty, there is only one path from *L* to Root. Combine the item-index and dosages in all nodes on the path with prefixes pf_1_′ and pf_2_′ to obtain corresponding level-1 and level-2 k-FWIs (1 ≤ *k* ≤ *K*), and let the WS of these new modes be equal to the WS of *L*. If the generated level-1 k-FWI, *β* already exists in the MRL, and the procedure Insert-MRL() is called to save *β* and the corresponding level-2 k-FWI, *γ* into the MRL. The execution process of Insert_MRL() is first determine whether the number of level-2 k-FWI corresponding to *β* is less than *n*, if it is, insert *γ* directly into the MRL; otherwise, if WS(*γ*) > *δ*_*k*_^2^(*β*), replace the level-2 k-FWI with the smallest WS of level-2 k-FWIs corresponding to *β* with *γ*. Finally, let *δ*_*k*_^2^(*β*) = the smallest WS of level-2 k-FWIs corresponding to *β*.
- If it is not empty, there are multiple paths to Root from the unit where *a*_*i*_ is saved. Combine item-index and dosage on a path into a conditional pattern, call the procedure Construct_MWP-tree(), and construct the conditional MWP-tree, CMT from these conditional patterns. Then, the process MWP-growth-2() is called iteratively with CMT, pf_1_′, and pf_2_′ as input parameters.

**Figure 7:**
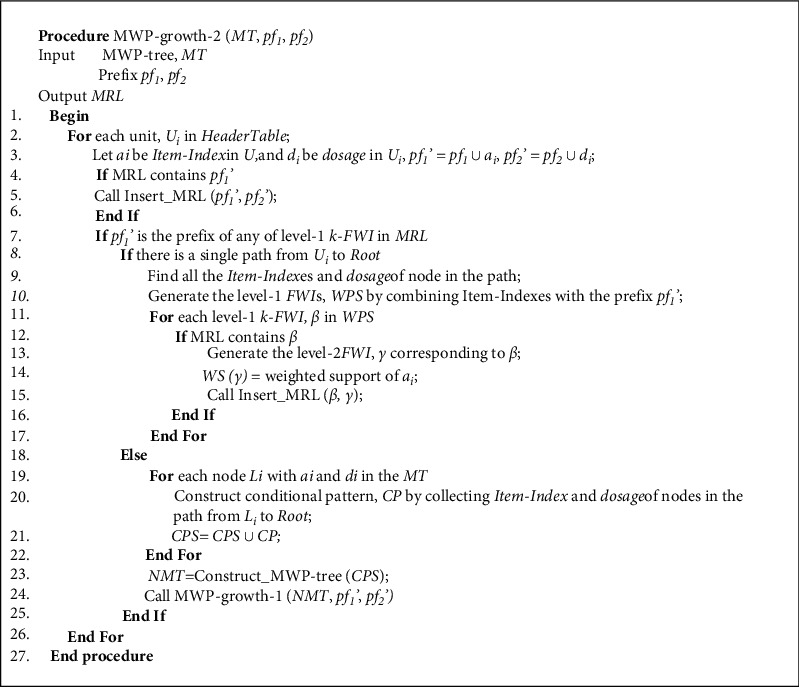
Algorithm for MWP-growth-2.

The pseudocode of MWP-growth-2() is shown in Figure 7.

**Example 4 .**

**Figure 8:**
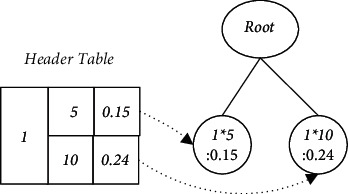
Condition tree example.

When *n* = 2, mine level-2 Top-K FWIS from the MWP-tree shown in Figure 4. Suppose Item-Index is *a*_*i*_, the unit of the HeaderTable whose dosage field value is *d*_*i*_ is marked as (*a*_*i*_ ∗ *d*_*i*_), there is only one path from the first unit (1 ∗ 5) to Root, and level-1 1-FWI can be obtained as {1 : 1}. The corresponding level-2 1-FWI is {5 : 0.56}. Similarly, from the second unit, we obtain level-1 1-FWI: {1 : 1} and another corresponding level-2 1-FWI: {10 : 0.44}. From the unit (2 ∗ 2), level-1 1-FWI: {2 : 0.7} corresponds to level-2 1-FWI: {2 : 0.39}, and since it has two paths to Root, its conditional pattern is <1 ∗ 5 : 0.15> and <1 ∗ 10 : 0.24>. The condition tree can be constructed, as shown in Figure 8. Thus, level-1 2-FWI: {2, 1 : 0.7} corresponds to level-2 2-FWI: {2, 5 : 0.15}, {2, 10 : 0.24}, and *δ*_*k*_^2^(2, 1 : 0.7) = 0.15. From the unit (2 ∗ 10), we can obtain the level-2 1-FWI: {10 : 0.31} corresponding to {2 : 0.7}. It has one path to Root, so we can obtain the level -2 2-FWI: {10, 5 : 0.31} corresponding to {2, 1 : 0.7}. Since the WS of level-2 2-FWI:{10, 5 : 0.31} is greater than *δ*_*k*_^2^(2, 1 : 0.7), it is used to replace {2, 5 : 0.15} in the MRL. With this method, all level-2 k-FWIs in MRL can be obtained, as shown in Table 3.

Finally, the level-1 k-FWI in MRL is transformed into a drug combination, where the pattern with the largest weighted support is the drug combination with the best efficacy, and the level-2 k-FWI with the highest weighted support is the optimal dosage of the drug combination. For instance, {3, 2, and 1: 0.55} can be transformed into a 3-drug combination: {Rehmannia glutinosa, Angelica sinensis, and Astragalus}, and the optimal doses of these three drugs are 7, 10, and 5 g, respectively.

## 4. Experiment Results

Literature on independent clinical studies of TCM treatment of osteoporosis in the Chinese language was retrieved from the CNKI, and then, eligible literature was screened. The selection criteria included the following: (1) randomized controlled trials (RCT) that were conducted in the study; (2) the patients were randomly divided into the control group and the treatment group. The control group was treated with conventional western medicine, such as taking vitamin D and calcium tablets, while the treatment group was treated with TCM; (3) the clinical treatment effects of the two patient groups were documented; (4) effective treatment means that the pain has been significantly reduced or eliminated, and bone density has increased or remained constant in comparison with before treatment. Invalid treatment means that the treatment resulted in no improvements or even worsening in all aspects when compared to before treatment.

First, the TCM prescriptions for treating osteoporosis are weighted according to the treatment effect, and then, the following is set: *K* = 5, *N* = 3, and *n* = 2. The MTWSR algorithm is used to mine prescription patterns with lengths from 1 to 5, which are listed in Table 4. For comparison, the same prescriptions are not weighted, and the traditional association rule algorithm Apriori is used for mining. The prescription patterns mined are listed in Table 5.

Rehmannia glutinosa has the highest support at 71.30%, which means it is the most commonly used drug. Eucommia ulmoides has the highest weighted support of 70.35, so it is the drug with the best treatment effect. When the dose of Eucommia ulmoides is 15 g, it has the best effect, with weighted support of 34.78. The most commonly used 2-drug combination is Eucommia ulmoides and Rehmannia glutinosa, which has a support rate of 49.57%. The best 2-drug combination is Angelica sinensis and Eucommia ulmoides with weighted support of 52.96. When the dose of Angelica sinensis is 10 g and the dose of Eucommia ulmoides is 15 g, the 2-drug combination has the best effect, with weighted support of 11.79. The most commonly used 3-drug combination is Angelica sinensis, Eucommia ulmoides, and Rehmannia glutinosa, and its support is 35.65. It is also the most effective 3-drug combination with a weighted support of 37.79. When the doses of these three drugs are 15, 15, and 30 g, the drug combination has the best effect, and the weighted support is 4.28. The most commonly used and most effective 4-drug combination is Epimedium brevicornu Maxim, Eucommia ulmoides, Rehmannia glutinosa, and Drynaria fortunei. The most commonly used 5-drug combination is Miltiorrhiza, Drynaria fortunei, Angelica sinensis, Epimedium brevicornu Maxim, and Rehmannia glutinosa with 17.39% support. The best 5-drug combination is Astragalus membranaceus, Angelica sinensis, Eucommia ulmoides, Cuscuta chinensis Lam, and Drynaria fortunei with a weighted support of 15.75. The optimal doses of these 5 drugs are 30, 10, 15, 10, and 15 g, and at this time, the 5-drug combination has the best effect, with a weighted support of 6.77.

## 5. Discussion

It can be seen from formula (1) that the larger the sample size of RCT, the better the treatment efficiency; the prescription used by the RCT is given a higher weight; and the drug combination contained in the prescription has a higher weighted support. This has led to the rising in the MRL rank of a combination of drugs that are not frequently used in TCMWTD but with better efficacy.

Clinical practice guidelines of traditional Chinese medicine for primary osteoporosis [16] recommend using Rehmannia glutinosa, Eucommia ulmoides, Angelica sinensis, Epimedium brevicornu Maxim, and other drugs to treat osteoporosis. Although Rehmannia glutinosa is the most commonly used medicine for osteoporosis in Table 5, Eucommia ulmoides has a higher weighted support than Rehmannia glutinosa, and it is the most effective medicine as shown in Table 4. This is because in RCT, the prescription of Eucommia ulmoides has a better effect than the prescription of Rehmannia glutinosa. For example, the RCT described in the literature [17] used a prescription containing Rehmannia glutinosa. The treatment result was that 24 of the 26 samples in the treatment group were effective, and 19 of the 26 samples in the control group were effective. The weight of the prescription is 57.5. In RCT [18], using a prescription containing Eucommia ulmoides at a dose of 12 g, the treatment result was that 48 out of 50 samples in the treatment group were effective, and 41 out of 50 samples in the control group were effective. Regarding the dosage of Eucommia ulmoides, 15 g is more effective than 12 g. For example, RCT [19] used Eucommia ulmoides at a dosage of 15 g and achieved better therapeutic effects than RCT [18]: 74 out of 82 samples in the treatment group were effective, and 63 out of 82 samples in the control group were effective. The weight of the prescription is 200.1.

With 2-drug combinations, although Eucommia ulmoides and Rehmannia glutinosa are used more frequently in RCT, the treatment efficiency of RCT with Angelica sinensis and Eucommia ulmoides is higher. For instance, after using Eucommia ulmoides and Rehmannia glutinosa in RCT [20], the treatment result is that 24 of the 26 samples in the treatment group are effective, and 19 of the 27 samples in the control group are effective. The weight of the prescription used is 53.2. RCT [21] used prescriptions containing Angelica sinensis and Eucommia ulmoides, and the treatment results were better: 28 of the 30 samples in the treatment group were effective, and 25 of the 30 samples in the control group were effective. 110.5 was the prescription weight. According to TCM theory [22], osteoporosis is caused primarily by a lack of kidney essence, qi, and blood, so treatment should focus on replenishing qi and blood, filling essence, and replenishing the marrow. Eucommia ulmoides is warm and belongs to the liver and kidney meridian. Angelica sinensis is sweet, pungent, and warm, returning to the liver, heart, and spleen meridian. The medicines listed above are used in combination to energize the liver and kidney, strengthen muscles and bones, increase blood circulation, remove dampness and dredge collaterals, and repair bones to heal injuries. Therefore, from the perspective of TCM theory, the 2-drug combination of Angelica sinensis and Eucommia ulmoides is also an ideal drug combination for the treatment of osteoporosis. In RCT [21], the doses of Angelica sinensis and Eucommia ulmoides are both 15 g, but when their doses are 10 and 15 g, respectively, the 2-drug combination has the best effect. For example, in RCT [23], their doses were 10 and 15 g, respectively, and the treatment result was that 45 of the 49 samples in the treatment group were effective, and 37 of the 49 samples in the control group were effective.

Similarly, although the frequency of occurrence is low, due to its good efficacy in RCT, the 5-drug combination of Astragalus membranaceus, Angelica sinensis, Eucommia ulmoides, Cuscuta chinensis Lam, and Drynaria fortunei has the highest weighted support, and the drug dosages of 30, 10, 15, 10, and 15 g have the best effect.

## 6. Conclusions

To efficiently mine TCM prescription patterns that include drug dosages, this paper proposed a multilevel weighted association rule algorithm MTWSR. The MTWSR algorithm first constructs the MWP-tree, then mines the level-1 Top-K FWI to get the drug combination with the best curative effect, and finally mines the level-2 Top-K FWI to get the best dosage of the drug combination. The experimental results show that MTWSR can be used to find drug combinations with less support but good curative effects, and these drug combination rules are effective from the perspective of TCM medical theory. These findings will aid doctors in prescribing appropriate drug combinations in clinical settings, as well as contribute to the modernization of TCM and further clinical research. In this study, MTWSR is used to mine prescription patterns from some prescriptions used in RCT. However, there are many more prescriptions and more detailed information about efficacy is recorded in clinical databases. In the future, we will mine more reliable prescription patterns from these clinical databases.

## Figures and Tables

**Table 1:** Examples of prescriptions in randomized controlled trials.

TID	Prescriptions
1	Astragalus∗5 g, Rehmannia glutinosa∗7 g, Angelica sinensis ∗10 g, Eucommia ulmoides ∗15 g, and Drynaria fortunei ∗5 g
2	Rehmannia glutinosa∗12 g, Eucommia ulmoides∗15 g, and Astragalus∗5 g
3	Astragalus∗10 g, Rehmannia glutinosa∗7 g, and Angelica sinensis∗2 g
4	Astragalus∗10 g and Eucommia ulmoides∗3 g
5	Astragalus∗5 g, Angelica sinensis∗2 g, and Drynaria fortunei∗10 g

**Table 2:** Treatment results of randomized controlled trials.

TID	*N*_1_	*E*_1_	*N*_2_	*E*_2_	Absolute weight	Weight (%)
1	100	91	100	82	314.1	0.31
2	41	38	41	31	102.1	0.10
3	65	62	65	53	236.5	0.24
4	60	55	60	50	206.3	0.20
5	65	59	65	49	151.8	0.15

**Table 3:** MRL after mining.

	Level-1 k-FWI	Level-2 k-FWI
1-FWI	1 : 1.0	5 : 0.56
		10 : 0.44
	2 : 0.7	2 : 0.39
		10 : 0.31
	3 : 0.65	7 : 0.55
		12 : 0.1

2-FWI	2, 1 : 0.7	10, 5 : 0.31
		2, 10 : 0.24
	3, 1 : 0.65	7, 5 : 0.31
		7, 10 : 0.24
	4, 1 : 0.61	15, 5 : 0.41
		3, 10 : 0.2

3-FWI	3, 2, 1 : 0.55	7, 10, 5 : 0.31
		7, 2, 10 : 0.24
	4, 3, 1 : 0.41	15, 7, 5 : 0.31
		15, 12, 5 : 0.1
	5, 2, 1 : 0.46	5, 10, 5 : 0.31
		10, 2, 5 : 0.15

**Table 4:** The mined multilevel Top-5 FWI.

	Level-1 frequent weighted patterns	Level-2 frequent weighted patterns
1-FWI	Eucommia ulmoides (70.35)	15 (34.78)
		12 (14.74)
	Rehmannia glutinosa (69.02)	15 (20.74)
		20 (18.90)
	Angelica sinensis (64.83)	10 (23.41)
		15 (20.99)

2-FWI	Angelica sinensis and Eucommia ulmoides (52.96)	10, 15 (11.79)
		15, 15 (10.97)
	Eucommia ulmoides and Rehmannia glutinosa (50.88)	15, 15 (10.75)
		15, 20 (7.20)
	Angelica sinensis and Rehmannia glutinosa (45.12)	15, 20 (5.36)
		15, 15 (4.87)

3-FWI	Angelica sinensis, Eucommia ulmoides, and Rehmannia glutinosa (37.79)	15, 15, 20 (4.28)
		15, 12, 15 (2.85)
	Angelica sinensis, Eucommia ulmoides, and Drynaria fortunei (30.89)	10, 15, 15 (6.77)
		10, 15, 10 (2.99)
	Epimedium brevicornu Maxim, Rehmannia glutinosa, and Drynaria fortunei (30.85)	15, 15, 15 (4.41)
		12, 15, 15 (3.74)

4-FWI	Epimedium brevicornu Maxim, Eucommia ulmoides, Rehmannia glutinosa, and Drynaria fortunei (21.90)	12, 15, 15, 15 (3.74)
		10, 15, 15, 10 (2.68)
	Astragalus membranaceus, Angelica sinensis, Eucommia ulmoides, and Drynaria fortunei (20.79)	30, 10, 15, 15 (6.77)
		20, 15, 12, 15 (2.26)
		15, 10, 15, 10 (2.26)
	Achyranthes bidentata Blume, Angelica sinensis, Eucommia ulmoides, and Rehmannia glutinosa (20.70)	15, 15, 12, 15 (2.26) 15, 15, 15, 20 (1.64)

5-FWI	Astragalus membranaceus, Angelica sinensis, Eucommia ulmoides, Cuscuta chinensis Lam, and Drynaria fortunei (15.75)	30, 10, 15, 10, 15 (6.77)
		15, 09, 12, 12, 09 (2.91)
	Epimedium brevicornu Maxim, Angelica sinensis, Miltiorrhiza, Rehmannia glutinosa, and Drynaria fortunei (14.82)	10, 10, 25, 15, 10 (2.96)
		15, 15, 15, 15, 15 (2.26)
	Epimedium brevicornu Maxim, Angelica sinensis, Eucommia ulmoides, Rehmannia glutinosa, and Drynaria fortunei (14.51)	15, 15, 12, 15, 15 (2.26)
		30, 12, 20, 20, 30 (1.51)

**Table 5:** The mined Top-5 most frequent patterns.

	Frequent patterns	Support(%)
1-FI	Rehmannia glutinosa	71.30
	Eucommia ulmoides	65.22
	Angelica sinensis	60.00
2-FI	Eucommia ulmoides and Rehmannia glutinosa	49.57
	Angelica sinensis and Eucommia ulmoides	45.22
	Angelica sinensis and Rehmannia glutinosa	44.35
	Epimedium brevicornu Maxim and Rehmannia glutinosa	44.35
3-FI	Angelica sinensis, Eucommia ulmoides, and Rehmannia glutinosa	35.65
	Epimedium brevicornu Maxim, Eucommia ulmoides, and Rehmannia glutinosa	31.30
	Achyranthes bidentata Blume, Eucommia ulmoides, and Rehmannia glutinosa	23.48
4-FI	Epimedium brevicornu Maxim, Eucommia ulmoides, Rehmannia glutinosa, and Drynaria fortunei	24.35
	Epimedium brevicornu Maxim, Angelica sinensis, Eucommia ulmoides, and Rehmannia glutinosa	21.74
	Epimedium brevicornu Maxim, Miltiorrhiza, Rehmannia glutinosa, and Drynaria fortunei	21.74
	Epimedium brevicornu Maxim, Angelica sinensis, Rehmannia glutinosa, and Drynaria fortunei	21.74
5-FI	Miltiorrhiza, Drynaria fortunei, Angelica sinensis, Epimedium brevicornu Maxim, and Rehmannia glutinosa	17.39
	Epimedium brevicornu Maxim, Angelica sinensis, Eucommia ulmoides, Rehmannia glutinosa, and Drynaria fortunei	16.52
	Epimedium brevicornu Maxim, Miltiorrhiza, Eucommia ulmoides, Rehmannia glutinosa, and Drynaria fortunei	15.65

## Data Availability

The data used to support the findings of this study are available from the corresponding author upon request.
